# Cocaine engages a non-canonical, dopamine-independent, mechanism that controls neuronal excitability in the nucleus accumbens

**DOI:** 10.1038/s41380-018-0092-7

**Published:** 2018-06-07

**Authors:** Ilse Delint-Ramirez, Francisco Garcia-Oscos, Amir Segev, Saïd Kourrich

**Affiliations:** Department of Psychiatry, University of Texas Southwestern Medical Center, Dallas, TX 75390 USA

**Keywords:** Neuroscience, Physiology

## Abstract

Drug-induced enhanced dopamine (DA) signaling in the brain is a canonical mechanism that initiates addiction processes. However, indirect evidence suggests that cocaine also triggers non-canonical, DA-independent, mechanisms that contribute to behavioral responses to cocaine, including psychomotor sensitization and cocaine self-administration. Identifying these mechanisms and determining how they are initiated is fundamental to further our understanding of addiction processes. Using physiologically relevant in vitro tractable models, we found that cocaine-induced hypoactivity of nucleus accumbens shell (NAcSh) medium spiny neurons (MSNs), one hallmark of cocaine addiction, is independent of DA signaling. Combining brain slice studies and site-directed mutagenesis in HEK293T cells, we found that cocaine binding to intracellular sigma-1 receptor (*σ*1) initiates this mechanism. Subsequently, *σ*1 binds to Kv1.2 potassium channels, followed by accumulation of Kv1.2 in the plasma membrane, thereby depressing NAcSh MSNs firing. This mechanism is specific to D1 receptor-expressing MSNs. Our study uncovers a mechanism for cocaine that bypasses DA signaling and leads to addiction-relevant neuroadaptations, thereby providing combinatorial strategies for treating stimulant abuse.

## Introduction

Enhanced dopamine (DA) signaling is postulated to be a canonical mechanism responsible for drug addiction [1, 2], but also an initial and sufficient event for the development of drug addiction. Previous studies suggest that activation of the sigma-1 receptor (*σ*1), an endoplasmic reticulum (ER) chaperone [3] that regulates a variety of proteins through physical protein–protein interactions [4], contributes to addiction processes [5, 6]. Consistent with this role, *σ*1 regulates both DA receptors signaling (DARs) via protein–protein interactions [7, 8] and DA release in the striatum [9]. In contrast, there is evidence that cocaine and other stimulants may also engage *σ*1 independent of DA signaling and contribute to cocaine addiction [10], suggesting that redundant or complementary mechanisms exist to shape addiction-related phenotypes. However, no cellular mechanism has been identified so far.

Previous studies showed that repeated in vivo exposure to cocaine leads to persistent neuronal hypoactivity in the nucleus accumbens shell (NAcSh) (i.e., firing rate depression, FRD)—an adaptation that enhances both psychomotor response to cocaine and cocaine reward [11]. Our previous study shows that cocaine-induced FRD is mediated by the activation of *σ*1 in the NAcSh and lasts up to 2 weeks after the last cocaine injection. Importantly, prior in vivo blockade of *σ*1 prevents both the development and the maintenance of cocaine-induced FRD in NAcSh medium spiny neurons (MSNs) [12], suggesting that the effect of *σ*1 blockade during cocaine treatment is enduring. This form of cocaine-driven intrinsic plasticity is now emerging as one of the hallmarks for cocaine addiction [13, 14]. Here, we demonstrate that cocaine-induced FRD is not prevented by DA receptor antagonists and is unaffected by a non-selective monoamine reuptake inhibitor, but is blocked by the *σ*1 antagonist BD1063. Combining in vivo pharmacology, biochemical and whole-cell patch-clamp studies on freshly dissected brain slices, with site-directed mutagenesis of *σ*1 expressed in HEK293T cells, we demonstrate that cocaine-*σ*1 physical interaction initiates the mechanism responsible for cocaine-induced FRD in NAcSh D1R-expressing MSNs. Further in vitro studies in brain slices demonstrate that, in contrast to typical drug actions on plasma membrane targets, cocaine initiates this mechanism by binding to *σ*1 intracellularly. Together, our results indicate that in addition to conventional mechanisms, psychostimulant drugs can also bypass DA signaling and lead to addiction-relevant neuroadaptations, and in particular, cocaine-driven plasticity of neuronal intrinsic excitability in NAcSh D1R-MSNs.

## Materials and methods

### Animals

Male C57BL/6J mice or male Drd1a-tdTomato C57BL6J mice (bred on site) (7–12 weeks of age). Mice were group housed and maintained on a 12-h light/dark cycle (light on at 7:00 a.m). See Supplementary Information for details. The experimental procedures followed the Guide for the Care and Use of Laboratory Animals (eighth edition) and were approved by the Animal Care and Use Committee at the University of Texas Southwestern Medical Center.

### Slice preparation and solutions

Sagittal slices of the NAcSh (250 µm) were prepared as described previously [11, 12, 15–17]. Slices recovered in artificial cerebro-spinal fluid (ACSF) saturated with 95% O_2_/5% CO_2_. See Supplementary Materials and methods for details.

### Electrophysiology

Whole-cell current-clamp recordings were performed as previously described [11, 12, 16]. See Supplementary Materials and methods for details.

### Cell culture and transfection

HEK293T cells were cultured at 37 °C and 5% CO_2_ in Dulbecco’s modified Eagle’s medium (DMEM, Invitrogen) without sodium pyruvate containing 10% fetal bovine serum with 100 mg/ml streptomycin sulfate, and 100 U/ml penicillin G sodium. Transfection of cells with expression vectors pCMV6-Kv1.2 (Cat. MC216959 OriGene) and pcDNA3.1-*σ*1-V5-His (kindly provided by Dr. Tsung-Ping Su) was done with Lipofectamine LTX DNA Reagent (Invitrogen) according to manufacturer’s instructions. Stable cell lines expressing *σ*1 or Kv1.2 were established using G418 selection. Cells stably expressing Kv1.2 were transiently transfected with *σ*1-V5 vector.

### Site-directed mutagenesis

The D188N mutations and the C-terminal 16 amino acids deletion of *σ*1 were introduced sequentially to the pcDNA3.1-*σ*1-V5-His vector using the Phusion Site-Directed Mutagenesis Kit (ThermoFisher) according to the manufacturer’s instructions.

### Immunoprecipitation from tissue and membrane isolation

Medial NAcSh was collected using the same procedure as described in “Slice preparation and solutions”. After recovery and drug treatment, 2–3 slices at a time were transferred to ice-cold ACSF and NAcSh were microdissected. For co-immunoprecipitation assays on tissue from in vivo cocaine-treated mice, NAcSh tissues were microdissected directly after slicing (as previously performed in [12]). See Supplementary Materials and methods for details.

### Statistics

Data acquisition and analysis were performed blind to experimental conditions when possible. Results are presented as mean ± S.E.M. Statistical significance was assessed using two-tailed Student’s *t*-tests, one-way analysis of variance (ANOVA) or two-way repeated-measures ANOVA and Bonferroni post-hoc tests when appropriate.

## Results

### Cocaine decreases NAcSh neuronal intrinsic excitability in D1R-expressing MSNs (D1R-MSNs)

**Fig. 1:**
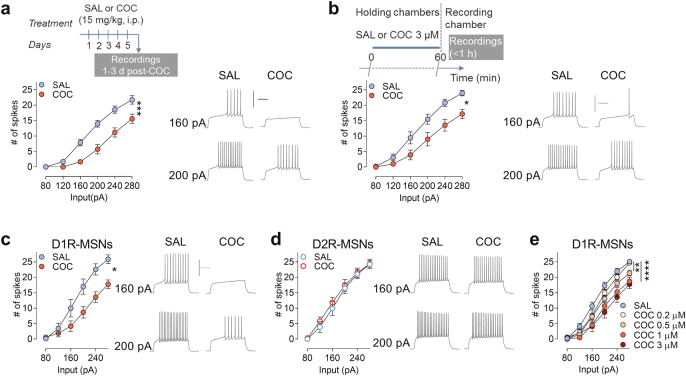
**Cocaine-induced FRD is specific to D1R-MSNs and is dose-dependent**. (**a**) Top: Experimental timeline. Bottom left: In vivo cocaine (five, once-daily i.p. injections at 15 mg/kg) decreases the number of spikes in NAcSh MSNs (brain slices) from cocaine-treated animals (SAL, saline, *n* = 10 cells/4 mice; COC, cocaine, *n* = 13 cells/4 mice). Bottom right: Sample traces. (**b**) Top: Experimental timeline. Bottom left: In vitro cocaine (COC, 3 μM, 1 h) decreases the number of spikes in NAcSh MSNs (brain slices) (SAL, *n* = 11 cells/5 mice; COC, *n* = 11 cells/5 mice). Bottom right: Sample traces. (**c, d**, left) Cocaine-induced FRD in cocaine-treated brain slices (COC, 3 μM, 1 h) occurs in D1R-MSNs (SAL, *n* = 12 cells/4 mice; COC, *n* = 15 cells/8 mice) (**c**) and not in D2R-MSNs (D2R-MSNs) (SAL, *n* = 9 cells/5 mice; COC, *n* = 13 cells/3 mice) (**d**). (**c, d,** right) Sample traces. (**e)** Cocaine-induced NAcSh MSNs FRD in D1R-MSNs is dose-dependent (SAL, *n* = 21 cells/8 mice; COC 0.2 μM, *n* = 11 cells/5 mice; COC 0.5 μM, *n* = 16 cells/5 mice; COC 1 μM, *n* = 9 cells/3 mice; COC 1 μM, *n* = 12 cells/8 mice). Because SAL groups from both panels (**c)** and (**e)** were similar, SAL data were combined. In (**a-e**), two-way ANOVA, ****p < 0.0001, ***p < 0.001, **p < 0.01, *p < 0.05. In (**e**), post-hoc tests: SAL group is different from all COC groups except COC 0.2 μM. Calibration: 200 ms, 50 mV. Data are represented as mean ± SEM

In vivo cocaine administration decreases NAcSh MSNs firing (Fig. 1a), consistent with previous studies that were performed in similar conditions, that is, without distinguishing MSN subtypes [DA 1 vs. DA 2 receptor-expressing MSNs, (D1R- and D2R-MSNs)] ([16, 18–20], reviewed in [13]). To determine its cellular mechanism, we developed a freshly dissected brain slice preparation that mimics in vivo physiological conditions. To this end, we applied cocaine in vitro at a concentration of 3 μM for 1 h, which corresponds to the concentration and half-life of cocaine in the NAc when injected i.p. at standard doses (10–20 mg/kg) [21, 22]. Using non-reporter C57BL/6J mice, we found that incubation of brain slices in cocaine (3 μM, 1 h) also depresses firing of NAcSh MSNs (Fig. 1b). Neuronal firing was assessed in cocaine-free ACSF at least 20’ after transferring slices to the recording chamber, which is sufficient to wash out cocaine [23]. To ensure that decreased firing rate is not due to blockade of voltage-gated Na^+^ currents (VGSCs) [24] by residual cocaine, we show that the low concentration of cocaine used (3 μM, 10 min) does not decrease Na^+^ currents (Supplementary Fig. S1a, b), consistent with previous studies [25]; or the action potential’s amplitude (Supplementary Fig. S1c), which is directly controlled by Na^+^ current.

Given the possible opponent roles of D1R- vs. D2R-MSNs in psychostimulant reward [26–28] and consistent with a previous study [29], we found that cocaine-induced FRD occurs specifically in D1R-MSNs (recorded from MSNs in Drd1a-tdTomato C57 mouse line, Fig. 1c) but not in D2R-MSNs (Fig. 1d). We also show that this effect is dose dependent (Fig. 1e, Supplementary Fig. S1d). Therefore, in subsequent studies, all recordings were conducted in D1R-MSNs in brain slices from Drd1a-tdTomato C57 mice.

Although cocaine-induced FRD is specific to D1R-MSNs, both our previous and others’ studies reliably obtained cocaine-induced FRD without distinguishing the two sub-populations of MSNs ([11, 12, 16, 18–20], reviewed in [13]). Although this may appear surprising, it is reminiscent of cocaine-induced synaptic potentiation in NAcSh neurons, that is, cocaine-induced synaptic plasticity in the NAcSh is specific to D1R-MSNs ([28], reviewed in [30]) but it is also obtained without distinguishing the two sub-populations of MSNs ([15], reviewed in [31, 32]). This effect may be explained by the inhomogeneous distribution of D1R- vs. D2R-MSNs in the NAcSh. Indeed, while studies have not specifically assessed neuronal subtype’s micro-distribution (potential existence of MSN subtype clusters) of D1R- and D2R-MSNs, both the distribution and the proportion of MSN subtypes are inhomogeneous. In particular, quantitative localization studies revealed the existence of D2R-MSN-poor zones [33] in the dorsomedial shell, near the subregion where our recordings were performed. Although qualitative but consistent with these findings, we show that while the total cell density in NAcSh appears homogenous, the rostral part of the medial shell presents a high density of D1R-MSNs (Supplementary Fig. S2). Another hypothetical and non-exclusive contributing factor is that cocaine also decreases neuronal firing in the 17% of NAcSh MSNs that co-express D1 and D2 receptors [34], allowing to obtain cocaine-induced FRD in the NAcSh consistently and reliably without distinguishing MSN subtypes.

### Cocaine-induced FRD is independent of DA or other monoamine signaling

The effect of DARs activation on the regulation of NAc MSNs firing is well documented (e.g., [35, 36]), and overactivation of DARs is a prominent hypothesis for cocaine-induced changes in the firing rate of NAc MSNs. However, studies show that coactivation of D1-like and D2-like receptors using DA or DAR agonists enhances MSNs firing in the NAcSh [35], which is opposite to what we have observed using cocaine in our previous and present studies [12, 16, 19, 20, 29] and by other groups [19, 20, 29] (reviewed in [13]). Altogether, these studies suggest that cocaine-induced FRD in NAcSh D1R-MSNs may involve additional or different mechanisms from DA signaling.

**Fig. 2:**
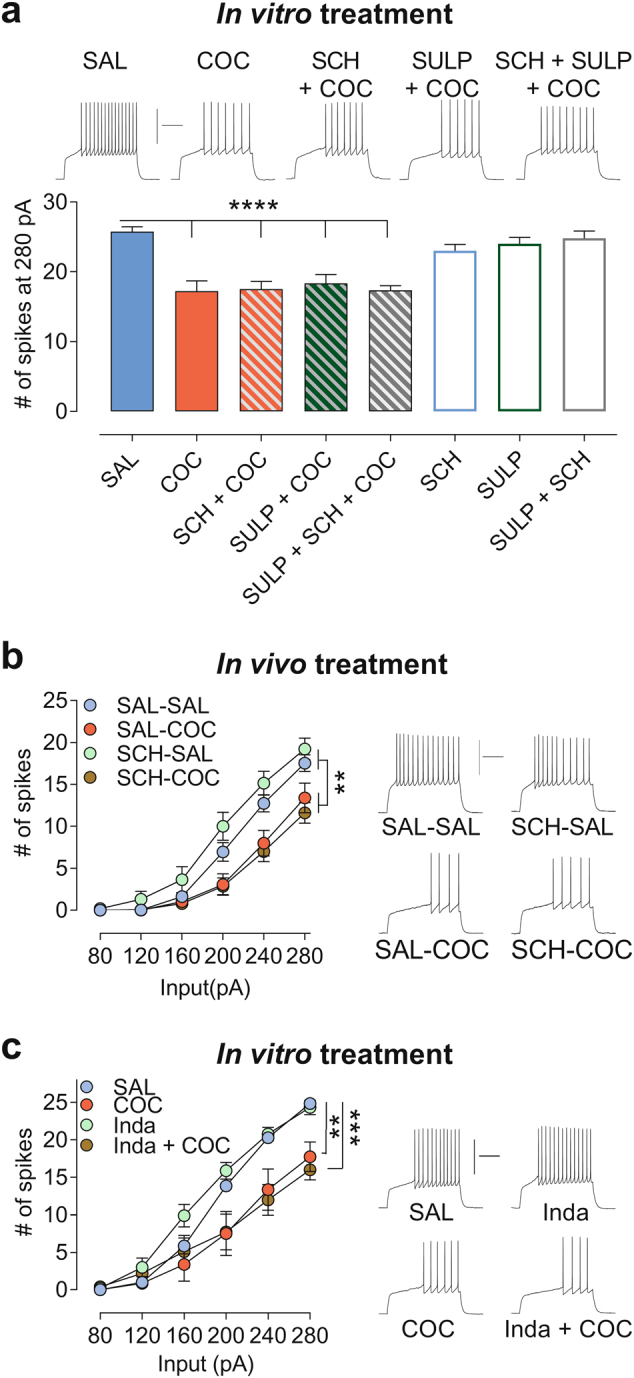
**Cocaine-induced FRD in NAcSh D1R-MSNs is neither prevented by DA receptor antagonists nor altered by a non-selective monoamine uptake inhibitor**. (**a**) Bottom: Summary bar graph showing the number of spikes elicited at 280 pA for all groups. Cocaine-induced FRD (COC, 3 μM, 1 h) is not blocked by DARs antagonists (D1R: SCH23390, SCH, 2 μM; D2R: Sulpiride, SULP, 10 μM). For the sake of visual clarity, we did not present the whole input–output curves but only the number of spikes at 280 pA, however, see Supplementary Fig. S3 for the complete curves (SAL, *n* = 17 cells/9 mice; COC, 9 cells/4 mice; SCH, 8 cells/3 mice; SCH + COC, *n* = 8 cells/3 mice; SULP, *n* = 12 cells/6 mice; SULP + COC, 9 cells/3 mice, SULP + SCH, 10 cells/3 mice; SULP + SCH + COC, 9 cells/4 mice). Top: Sample traces. (**b**) Left: Prior in vivo administration of SCH (0.01 mg/kg, i.p) does not prevent in vivo cocaine-induced FRD (15 mg/kg, i.p., five once-daily) (SAL-SAL, *n* = 18 cells/4 mice; SAL-COC, 15 cells/4 mice; SCH-SAL, 16 cells/4 mice; SCH + COC, *n* = 17 cells/4 mice). Right: Sample traces. (**c**) Left: Indatraline (Inda, 30 nM) does not alter firing rate in NAcSh MSNs, and cocaine-induced FRD remains unaffected by Inda (SAL, *n* = 7 cells/4 mice; COC, 8 cells/3 mice; Inda, 9 cells/4 mice; Inda + COC, *n* = 10 cells/3 mice). Right: Sample traces. In (**a**), one-way ANOVA, ****p < 0.0001. Post-hoc tests showed that none of the groups with COC is different from one another other; and that all other groups (SAL, SCH, SULP, and SCH + SULP) were different from COC but not different from one another other. ****p < 0.0001. In (**b**), two-way ANOVA, ***p < 0.001. Post-hoc tests showed that SAL-SAL is not different from SCH-SAL group, but both SAL-SAL and SCH-SAL are different from SAL-COC and SCH-COC. **p < 0.001. In **c**, two-way ANOVA, *p* < 0.05. Post-hoc tests showed that SAL is different from groups with cocaine, but not different from indatraline. **p < 0.01, ***p < 0.001. Calibration: 200 ms, 50 mV. Data are represented as mean ± SEM

In addition to inhibiting DA reuptake [1, 2], cocaine is also an agonist of *σ*1 [3] (reviewed in [37]), and both DARs [38] and *σ*1 regulate VGICs (reviewed in [39–41]), which suggest that cocaine may alter VGICs via both DAR- and *σ*1-dependent pathways. However, the relative contributions of *σ*1- and DA-dependent pathways in cocaine-induced changes in neuronal intrinsic excitability, and especially in NAcSh D1R-MSNs, is not known. Therefore, to determine whether cocaine-induced FRD in the NAcSh is underpinned by a non-canonical, DA-independent mechanism, we incubated brain slices in DAR antagonists prior to cocaine. We show that prior application of D1- (SCH23390, SCH, 2 μM) or D2-like receptor antagonists (Sulpiride, SULP, 10 μM) alone or combined does not prevent cocaine-induced FRD (Fig. 2a, Supplementary Fig. S3). Application of these antagonists alone or together does not alter basal firing rate (Fig. 2a, Supplementary Fig. S3d). To support these results further, if DAR-modulated VGICs also participate significantly to cocaine-induced FRD in NAcSh D1R-MSNs, we expect DAR blockade prior to cocaine to dampen the effect of cocaine on neuronal firing. Here, we demonstrate that cocaine alone or with D1R- and D2R-like antagonists decreases neuronal firing to the same extent (Fig. 2a). However, it is interesting to note that prior application of D1- (SCH23390, SCH, 2 μM) and D2-like receptor antagonists (SULP, 10 μM) slightly dampens cocaine-induced FRD at low depolarizing current injection (Supplementary Fig. S3c), however, this effect is not significant. As application of these antagonists together does not alter basal firing rate (Fig. 2a, Supplementary Fig. S3d), these data suggest that while cocaine-induced DA modulation of VGICs may occur, these VGICs do not seem to impact cocaine-induced FRD in NAcSh D1R-MSNs significantly.

As cocaine selectively decreases neuronal firing in D1R-MSNs, we also assessed the effect of the D1-like receptor antagonist administered in vivo (SCH, 0.1 mg/kg, i.p.) prior to cocaine (15 mg/kg, five once-daily) and at a dose that also abolishes acute psychomotor stimulant effects of cocaine. We found that in vivo SCH does not prevent cocaine-induced FRD in NAcSh D1R-MSNs (Fig. 2b).

As cocaine also blocks norepinephrine (NET) and serotonin transporters (SERT) and norepinephrine and serotonin signaling also modulate K+ currents and intrinsic excitability [42–44], we assessed the effect of a non-selective and structurally different general monoamine uptake blocker on NAcSh MSNs firing (indatraline, a.k.a. Lu 19-005). Indatraline (30 nM, 1 h), at a concentration significantly higher than its IC_50_ at the monoamine transporters (≈ 0.2–5 nM) [45–47], does not induce MSNs FRD on its own, or block cocaine-induced FRD (Fig. 2c). Altogether, these results are of particular importance as they provide direct evidence that cocaine mediates some of its addiction-relevant neuroadaptations via a non-canonical DA-independent mechanism (and likely NE- and 5-HT-independent).

### Cocaine binding to *σ*1 initiates the mechanism responsible for FRD in NAcSh D1R-MSNs

**Fig. 3:**
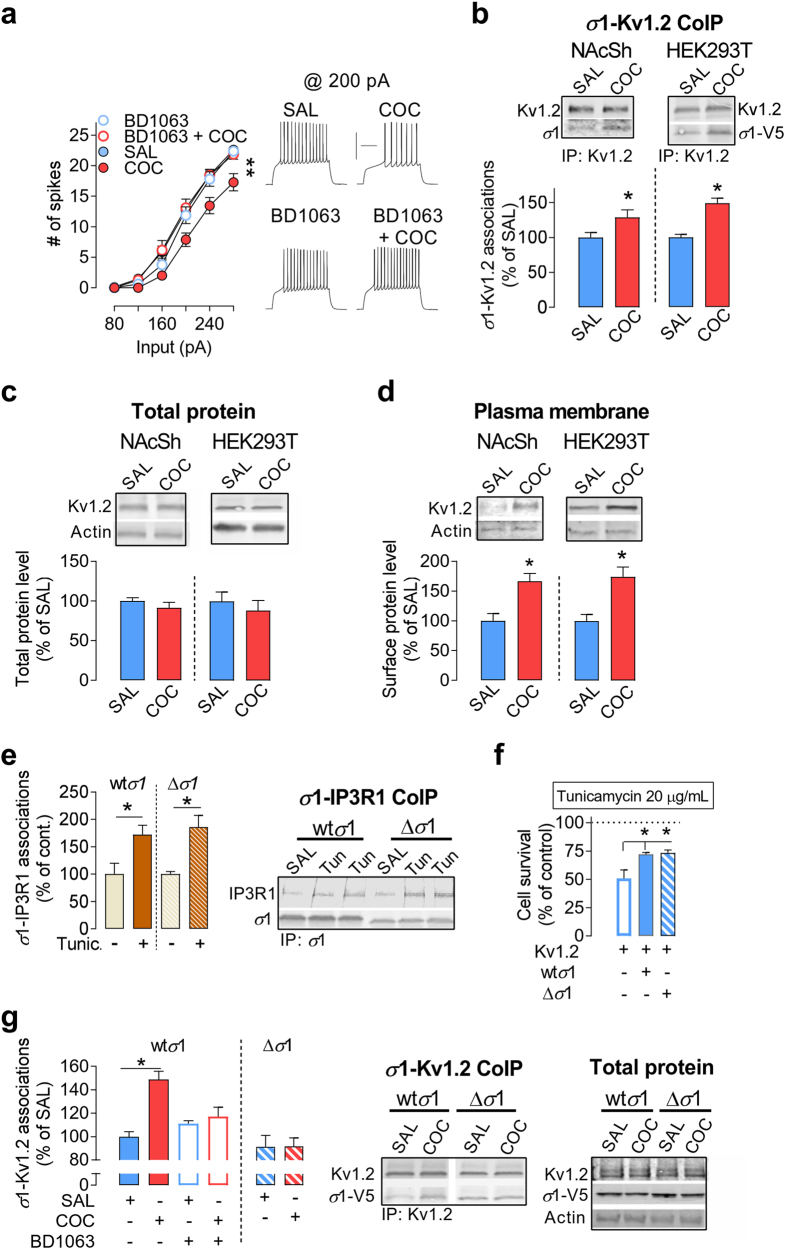
**Cocaine binding to** ***σ*1 enhances** ***σ*1-Kv1.2 interactions.** (**a**) Left: COC-induced FRD is prevented by prior bath application of BD1063 (500 nM, 20-25 min prior to COC). Right: Sample traces from NAcSh MSNs (SAL, *n* = 36 cells/7 mice; COC, 16 cells/6 mice; BD1063 + COC, 12 cells/3 mice; BD1063 + SAL, *n* = 8 cells/3 mice). (**b**) Cocaine (3 μM, 1 h) enhances *σ*1-Kv1.2 CoIP in both NAcSh and HEK293T cells. NAcSh: 8 samples/group, 4 mice/sample. HEK293T cells: five independent samples/group. Cell lysates were immunoprecipitated with the anti-Kv1.2 antibody; immunoprecipitated proteins were analyzed by western blot using anti-Kv1.2 (upper), and anti-*σ*1 antibody for brain samples or anti-V5 to detect *σ*1 in HEK293T cells (lower). (**c**) Kv1.2 total protein levels remained unchanged in both preparations, NAcSh tissue (SAL, 6 samples/group; COC, 8 samples/group; 4 mice/sample) and HEK293T cells (4 independent samples/group). Samples were analyzed by western blot. (**d**) Cocaine enhances Kv1.2 surface levels in NAcSh. Isolation of plasma membrane was performed with immobilized concanavalin A (ConA) magnetic beads (5 samples/groups, 10 mice/sample). Samples were analyzed by western blot. Right: Plasma membrane was isolated from HEK293T cells. Cocaine increases Kv1.2 in the plasma membrane (3 independent samples per group). (**e**) HEK293T cells stably overexpressing IP3R1 and wt*σ*1-V5 or Δ*σ*1-V5 were treated 2 h with tunicamycin (20 μg/mL) and immunoprecipitated with anti-V5 antibody; immunoprecipitated proteins were analyzed by western blot using anti-IP3R1 and anti-V5 antibody (wt*σ*1, cont, four independent samples; wt*σ*1, Tunic, six independent samples; Δ*σ*1, cont, four independent samples; Δ*σ*1, Tunic, five independent samples). (**f**) Both wt*σ*1 and Δ*σ*1 protect against tunicamycin-induced cell death. HEK293T cells stably express Kv1.2 alone, Kv1.2 with wt*σ*1, or Kv1.2 with Δ*σ*1 were treated overnight with tunicamycin (20 μg/mL) (Kv1.2, four independent samples; wt*σ*1 + Kv1.2, five independent samples; Δ*σ*1 + Kv1.2, four samples). Graph shows percentage of cell alive compare with cells treated with vehicle (DMSO). (**g**) COC (3 μM, 1 h) upregulates *σ*1-Kv1.2 CoIP in Kv1.2 stable HEK293T cells that overexpress wt*σ*1-V5, but not in cells that overexpress Δ*σ*1-V5. Note that blocking *σ*1 with BD1063 also prevents cocaine-induced increase in *σ*1-K1.2 complexes. Results are normalized to their respective SAL group (wt*σ*1: five independent samples per group; Δ*σ*1: SAL, eight independent samples; COC, seven samples). Note that due to truncation of the last 16 amino acids in Δ*σ*1, the band is slightly lower than wt*σ*1, and as expected. In (**a),** two-way ANOVA, **p < 0.01; post-hoc tests showed that SAL is different from COC, but not different from BD1063 and BD1063 + COC. In (**b**) and (**d**), Unpaired t-test, *p < 0.05. In (**e**) and (**g**), One-way ANOVA, *p < 0.05. Post-hoc tests: *p < 0.05. In **f,** Unpaired *t*-test, *p < 0.05. Calibration: 200 ms, 50 mV. Data are represented as mean ± SEM

Prior bath application of the prototypical *σ*1 antagonist BD1063 (500 nM), at a concentration that does not alter basal firing rate, prevents cocaine-induced FRD (Fig. 3a). Cocaine is an agonist of *σ*1 [3] (reviewed in [37]) and has been shown to alter several sodium, calcium and potassium conductances in NAc MSNs, each of which is consistent with a decrease in depolarization-induced firing [18, 19, 48–50]. These data raise the hypothesis that cocaine binding to *σ*1 is the initial event that leads to the regulation of VGICs responsible for the FRD in NAcSh D1R-MSNs. However, although other conductances are altered upon cocaine exposure, our previous electrophysiological and pharmacological studies suggest that they do not contribute significantly to cocaine-induced FRD [12]. Furthermore, combining electrophysiological, pharmacological and biochemical analyses, we show that in vivo cocaine depresses neuronal firing via a mechanism that critically involves the formation of *σ*1-Kv1.2 protein complexes and upregulation of Kv1.2 K+ channels at the plasma membrane of NAcSh MSNs [12]. Although we do not exclude the relative participation of other conductances in cocaine-induced FRD, to determine whether cocaine-induced FRD is triggered by cocaine binding to *σ*1, we developed a cell culture model that recapitulates a critical biochemical outcome that underlies cocaine-induced FRD in vivo, that is, enhanced *σ*1–Kv1.2 interactions (Supplementary Fig. S4a and [12]). First, we show that cocaine enhances co-immunoprecipitation (CoIP) of *σ*1 with Kv1.2 in both freshly dissected brain slices (Fig. 3b, left) and HEK293T cells (Fig. 3b, right, Supplementary Fig. S4b). To determine whether the increase in *σ*1–Kv1.2 interactions correlates with recruitment of Kv1.2 to surface, we isolated plasma membranes from NAcSh tissue and HEK293T cells using immobilized Concanavalin A magnetic beads [51–53] (Supplementary Fig. S4c), and found that cocaine enhances surface levels of Kv1.2 (Fig. 3d), whereas total protein levels of Kv1.2 α-subunits and *σ*1 remain unchanged (Fig. 3c). These data also suggest that cocaine-induced upregulation of *σ*1–Kv1.2 complexes and surface Kv1.2 are a conserved cellular mechanism that extend to non-neuronal heterologous systems. Therefore, we will use cocaine-induced increase in *σ*1–Kv1.2 complexes as readout for cocaine-induced activation of *σ*1.

Site-directed mutagenesis studies showed that cocaine binding to *σ*1 requires an aspartate residue at AA 188 located near the C-terminus, and the last 16 amino-acid residues of *σ*1 [54, 55]. Therefore, we truncated *σ*1 from the last 16 residues and replaced Asp188 by Asn188 (Δ*σ*1-V5), then overexpressed Δ*σ*1-V5 in HEK293T cell line along with Kv1.2 subunits. To verify otherwise competent chaperone capability of Δ*σ*1-V5, we first tested whether Δ*σ*1-V5 can associate with one of *σ*1’s targets IP3 receptors (IP3Rs) [56], and whether *σ*1–IP3R protein complexes are dynamically regulated by ER stress, a known mechanism for *σ*1 [3]. We found that tunicamycin-induced ER stress enhances associations of IP3R1 with both wt*σ*1 (native form of *σ*1) and Δ*σ*1-V5 (Fig. 3e). As *σ*1 exhibits protective properties against cell death [57–59], we also ensured that the Δ*σ*1-V5 protective effect is preserved. We found that while tunicamycin-induced ER stress leads to 50% cell death in cells overexpressing Kv1.2 alone, Δ*σ*1-V5 protected cells from tunicamycin-induced apoptosis to the same extent as wt*σ*1 (Fig. 3f). Although *σ*1 is an inter-organelle signaling modulator that exerts several distinct functions (e.g., ER lipid metabolisms/transports [60], and indirectly regulating the transcription of genes [4]), these data demonstrate that the two necessary functions to test our hypothesis, chaperone activity and protection against cell death, are preserved in Δ*σ*1-V5.

Significantly, although cocaine upregulates wt*σ*1–Kv1.2 complex levels, it fails to upregulate the formation of Δ*σ*1-Kv1.2 protein complexes with the mutant *σ*1 (Fig. 3g), similar to the effect of *σ*1 blockade with BD1063 (500 nM). This suggests that cocaine binding to *σ*1 is a necessary mechanism for the recruitment of additional *σ*1–Kv1.2 protein complexes.

### Cocaine-induced FRD is mediated via activation of intracellular *σ*1

*σ*1 is enriched in intracellular organelles, and especially at the ER level. Protonated cocaine, like several drugs including antidepressants and abused substances [61], coexists with their deprotonated form in the physiological milieu (membrane permeant), indicating that cocaine can cross the plasma membrane. In addition, although it is still a subject of research, it is thought that *σ*1 can also be inserted in the plasma membrane. Although the bulk of the C-terminus (containing the binding pocket) may be located either in the cytosol (crystal study in micelles) [62] or in the extracellular space (in vivo study in dorsal root ganglions) [63], Ruoho and colleagues demonstrate that inactive intracellular oligomeric states can bind the *σ*1 agonist (+)-pentazocine in vitro, and not monomer/dimer states that may exist at the membrane ([64], reviewed in [65]). Altogether, this suggests that cocaine targets intracellular *σ*1.

**Fig. 4:**
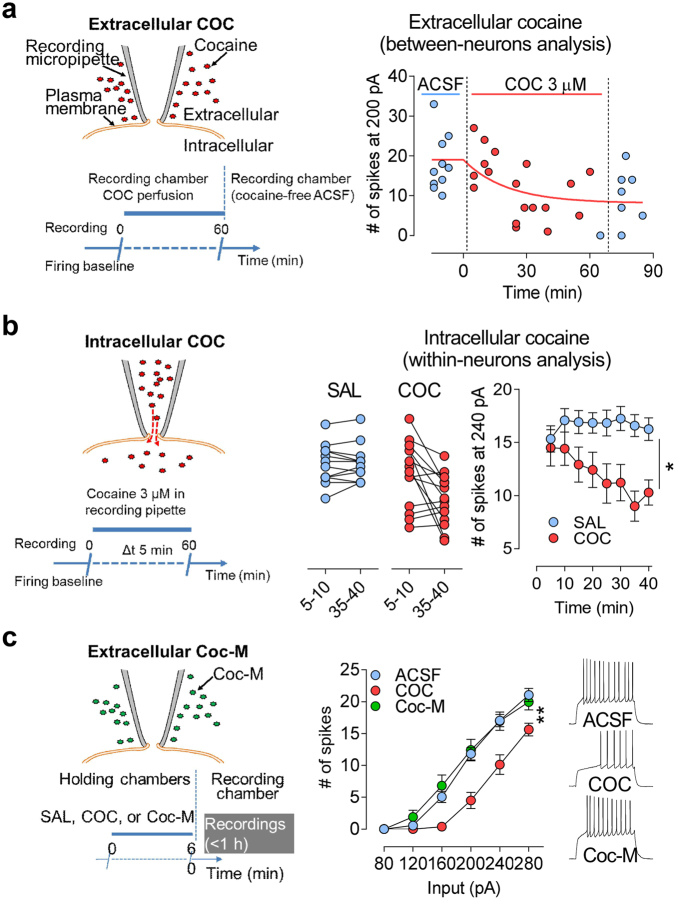
**Cocaine-induced FRD in D1R-MSNs is initiated by cocaine binding to intracellular** ***σ*1**. (**a**) Left: Scheme depicting extracellular bath perfusion of cocaine and experimental design. Right: Cocaine (3 μM) bath perfusion in the recording chamber decreases firing rate elicited with 200 pA current injection within 30 min. Neuronal firing was recorded before, during and after cocaine. Each data point represents individual neurons obtained from different slices and different mice (*n* = 36 cells/8 mice). Nonlinear regression shown that cocaine-induced FRD fit an exponential with one phase decay. *R*^2^ = 0.3101. (**b**) Left: Scheme depicting the strategy used to introduce cocaine inside neurons and experimental design. Right: Cocaine (3 μM) or saline (vehicle) were added to K-gluconate internal solution (5 μl/1000 μl). Right: Firing rate of individual neurons at 5–10 and 35–40 min after the establishment of whole-cell configuration. In far right, the mean number of spikes elicited at 240 pA for neurons recorded with micropipettes containing cocaine is decreased compared with vehicle (SAL) (SAL, *n* = 12 cells/7 mice; COC, *n* = 14 cells/9 mice). (**c**) Left: Scheme depicting extracellular bath perfusion of cocaine methiodide (Coc-M) and experimental design. Right: COC (3 μM, 1 h), but not Coc-M (3.9 μM, 1 h), decreases NAcSh D1R-MSNs (SAL, *n* = 17 cells/5 mice; COC, *n* = 8 cells/3 mice; Coc-M, *n* = 11 cells/3 mice). Right: Sample traces. In (**b**), Two-way ANOVA: treatment, *p<0.05; interaction, **p < 0.01. In (**c**), two-way ANOVA: treatment, **p < 0.01; interaction, *p* < 0.0001. Post-hot tests: ACSF is different from COC (*p* < 0.0001), but not Coc-M group. Calibration: 200 ms, 50 mV. Data are represented as mean ± SEM

To determine whether cocaine activating intracellular *σ*1 is the initiating mechanism for FRD in NAcSh D1R-MSNs, we introduced cocaine directly into the recording pipette. Beforehand, and to make accurate predictions on the onset of cocaine-induced FRD in D1R-MSNs, we perfomed a between-cell analysis with cocaine applied extracellularly and found that neuronal firing is decreased within ≈30 min from cocaine application (Fig. 4a, Supplementary Fig. S5a). Second, we introduced cocaine into the recording patch pipette and performed a within-neuron comparison. We analyzed spike trains elicited at a nonsaturating current injection of 200 pA or 240 pA, and found that intracellular cocaine decreases NAcSh D1R-MSNs firing within ~30 min after establishing whole-cell configuration (Fig. 4b, Supplementary Fig. S5a). Next, using (–)-cocaine methiodide (Coc-M), a chemical analog of cocaine with a stable positive charge at physiological pH that prevents free diffusion through membranes [66], we found that extracellular Coc-M at an equimolar concentration (3.9 μM) fails to decrease NAcSh D1R-MSNs firing (Fig. 4c, Supplementary Fig. S5b).

Taken together, these results obtained from complementary strategies provide direct evidence that cocaine depresses D1R-MSNs firing rate via its action on intracellular *σ*1.

## Discussion

Using in vivo and in vitro models, we provide convergent evidence that cocaine-induced hypoactivity of NAcSh D1R-MSNs is mediated by a DA-independent mechanism and is neither induced nor blocked by accumulation of monoamines in the synaptic cleft (Fig. 2 and Supplementary Fig. S3). Second, using site-directed mutagenesis of *σ*1 cocaine binding site in HEK293T cells, and both extracellular cocaine methiodide (non-permeant cocaine) and intracellular application of cocaine in brain slices, we demonstrate that cocaine-induced FRD is triggered by cocaine binding to intracellular *σ*1 (Fig. 3g and Fig. 4). Determining the mechanism by which cocaine crosses the plasma membrane, free diffusion or transported via an unidentified target, is beyond the scope of this study. Altogether, our study provides direct evidence that besides actions mediated through conventionally studied mechanisms, cocaine also engages a mechanism that is DA- independent, but *σ*1 binding-dependent. This mechanism of action leads to neuronal hypoactivity of NAcSh MSNs firing—an adaptation that promotes behavioral responses to cocaine (reviewed in [13]).

Although a previous study [29] and the present found that cocaine-induced FRD occurs specifically in D1R- but not in D2R-MSNs (Fig. 1c, d), these findings are unexpected. This suggests that other neuronal subtype-specific factors may control *σ*1-dependent functions (reviewed in [40, 41]). Further investigations are warranted to identify these factors. Although we hypothesize that a differential expression of targets of interests (i.e., *σ*1 and Kv1.2) is a factor, there is no study to date suggesting different levels of *σ*1 and Kv1.2 proteins in D1R- vs. D2R-MSNs.

### Non-canonical DA-independent mechanism triggered by psychostimulant drugs

Earlier studies show a clear implication of DA signaling in the acute locomotor stimulatory effects of cocaine, the gradual increase in cocaine-induced locomotion upon repeated cocaine treatment [67–70], and in cocaine self-administration (reviewed in [71]). However, several studies using various experimental designs to assess behavioral sensitization to cocaine in mice and rats showed that in vivo blockade of D1- or D2-like receptors, at doses that abolish acute psychomotor stimulant effects of cocaine, just attenuate or fail to prevent the induction of behavioral sensitization to cocaine, that is, enhanced psychomotor behavior when animals are challenged during withdrawal [72–74]. In addition, other studies suggest that the effect of systemic D1-like receptor antagonists on behavioral sensitization may also depend on cocaine doses used when animals are challenged [75]. Together, these studies imply that psychostimulant-induced DA signaling is important in the development of addiction-related behaviors; however, DA signaling may be differentially involved as a function of experimental conditions, the behavioral paradigm used, or the behavioral stage under consideration (e.g., induction or expression of psychomotor sensitization). Therefore, cocaine and other stimulants may engage additional mechanisms that would participate in specific addiction-related phenotypes. In that regard, a stream of studies demonstrates that cocaine also engages mechanisms that are dependent on *σ*1, but independent of DA signaling, and which contribute to cocaine addiction [10]. For example, animals with cocaine experience, but not after experience with food reinforcement, self-administer *σ*1 agonists (e.g., PRE-084 and (+)-Pentazocine) [76] at doses that do not induce DA release in the NAcSh [77] (reviewed in [10]). Furthermore, self-administration of PRE-084 is blocked by *σ*1 antagonists (e.g., BD1063), but not blocked by the D1R antagonist SCH 39166 effective against cocaine ([76], reviewed in [10]). These data demonstrate that the role of DA in behavioral response to cocaine or the development of addiction-relevant behaviors is complex and that cocaine engages additional mechanisms that also participate in the development of addiction-relevant phenotypes.

Thus, the role of *σ*1 vs. DARs in cocaine’s behavioral effects remains elusive. Their contributions are likely synergetic, and teasing apart their relative contributions in cocaine-related behaviors and in specific stages of the addiction cycle (acquisition, extinction, relapse) has been an intense subject of research in Dr. Katz’s laboratory (reviewed in [10]). In the present study, we identify a DA-independent cellular mechanism by which cocaine alters neuronal intrinsic excitability (i.e., neuronal firing) of NAcSh MSNs.

Non-canonical, intracellular, actions of psychiatric drugs or abused substances are emerging as intriguing complementary mechanisms that contribute to their pernicious addictive properties [61]. For example, besides conventional action of nicotine on membrane nicotinic acetylcholine receptors (nAChRs), nicotine exhibits pharmacological chaperoning activity upon binding to specific intracellular nAChRs located at the ER level, which underlies some initial events of nicotine addiction (reviewed in [61]). Directly pertinent to the present study, intravenous self-administration of methamphetamine (METH) in rats, a psychostimulant drug that is chemically different from cocaine, decreases NAcSh MSNs firing rate [78]. METH also binds to *σ*1 [79], consistent with the capability of the *σ*1 ligand-binding cavity to bind structurally different compounds [37, 62]. Future studies are warranted to determine whether NAcSh neuronal hypoactivity is a unifying *σ*1-dependent, DA-independent, mechanism among abused psychostimulant drugs that bind *σ*1.

## Conclusion

Present and future information obtained on DA-independent, but *σ*1-dependent, mechanisms will have the potential to pave the way to novel and combinatorial pharmacotherapies to specifically treat stimulant abuse or provide alternatives for treatment-resistant stimulant abuse. Furthermore, because this mechanism of action occurs in cell type-specific manner (D1R- vs. D2R-MSNs), it suggests that the diversity of *σ*1’s effects on cellular physiology is influenced by *σ*1’s differential engagement of multiple signaling pathways that may depend on several biological factors. The translational implication of such findings is important; it suggests that directly targeting *σ*1 may have less unwanted side effects than originally expected. More fundamentally, the present findings also further our understanding of the mechanisms through which *σ*1 regulates K+ channel trafficking—a topic of broad neuroscientific and clinical interest.

## Electronic supplementary material

Supplementary Information
