# Supplementary material for: Cocaine engages a non-canonical, dopamine-independent, mechanism that controls neuronal excitability in the nucleus accumbens
Source: Mol Psychiatry. 2018 Jun 7;25(3):680–91. doi: 10.1038/s41380-018-0092-7 (PMC7042730; doi:10.1038/s41380-018-0092-7)
Supplement: Supplementary file 1 — Supplementary Information [file 41380_2018_92_MOESM1_ESM.docx]

**SupplementaRY INFORMATION**

**Cocaine engages a non-canonical, dopamine-independent, mechanism that controls neuronal excitability in the nucleus accumbens**

Ilse Delint-Ramirez, Ph.D.^1^ Francisco Garcia-Oscos, B.S.^1^ Amir Segev, Ph.D.^1^ and Saïd Kourrich Ph.D.^1*^

^1^Department of Psychiatry, University of Texas Southwestern Medical Center, Dallas, TX 75390, USA

Corresponding Author:

Saïd Kourrich, Ph.D.

University of Texas Southwestern Medical Center

Department of Psychiatry

Kourrich Lab NC6.206

2201 Inwood Road

Dallas, TX 75390-9070

Tel: (214) 648 1338 (office)

[Said.Kourrich@UTSouthwestern.edu](mailto:Said.Kourrich@UTSouthwestern.edu)

**Running title:**  Non-canonical mechanism of cocaine

**Drug Treatment Regimen**

To assess neuronal firing or *σ*1-Kv1.2 protein-protein interactions after a chronic cocaine regimen (Figure 1a, Supplementary Figure S4), on each of the five consecutive testing days (between 10:00 A.M. and 2:00 P.M.), cages were put on a cart and mice were injected i.p. in their home cage either with saline (0.9% NaCl) or cocaine (15 mg/kg) at a volume of 10 ml/kg. To mitigate any potential effects of stress, mice were handled, injected with saline (twice, one per day) and habituated to the experimental conditions before any drug treatment.

**Drugs**

Cocaine hydrochloride (Sigma-Aldrich) was dissolved in 0.9% NaCl. (S)-(−)-Sulpiride (Sigma-Aldrich), R(+)-SCH-23390 hydrochloride (Sigma-Aldrich), 1-[2-(3,4-dichlorophenyl)ethyl]-4-methylpiperazine (BD1063, Tocris), tetrodotoxin (TTX) (Tocris), indatraline hydrochloride (Sigma-Aldrich), and cocaine methiodide (provided by the National Institute on Drug Abuse) were dissolved in 0.9% NaCl and bath applied at indicated concentrations. Tunicamycin was dissolved in DMSO (final concentration was < 0.05 %).

**Slice Preparation and Solutions**

Cells were visualized using infrared-differential interference contrast optics. Medium spiny neurons were identified by their morphology and hyperpolarized resting membrane potential (-75 to -85 mV) and as previously described ([1-3](#_ENREF_1)). In a small number of cases, some cells showed clear electrophysiological signature of interneurons ([4-6](#_ENREF_4)) . These cells were excluded from further investigation. In studies involving Drd1a-tdTomato C57BL6J mouse line, D1R-expressing MSNs were identified by their fluorescence. MSNs that were not fluorescent and were not exhibiting the above characteristics were considered D2R-expressing MSNs. We examined neurons in medial NAcSh slices that did not contain dorsal striatal tissue (~0.44–0.52 mm lateral) (Supplementary Figure S2 right) ([7](#_ENREF_7)).

**Electrophysiology**

Brain slices recovered in a holding chamber for at least 1 h before use. ACSF contained (in mM) 119 NaCl, 2.5 KCl, 1.0 NaH2PO4, 1.3 MgSO4, 2.5 CaCl2, 26.2 NaHCO3 and 11 glucose. During recording slices were superfused with ACSF (31.5–32.5°C) saturated with 95% O_2_/5% CO_2_ and containing picrotoxin (100 μM) and kynurenic acid (2 mM) to block GABA_A_ receptor-mediated IPSCs and glutamate receptors respectively. Cocaine (Sigma-Aldrich) was either bath applied during recordings, introduced in the recording pipette solution, or administered in the incubation chambers. Indatraline was administered for at least one hour in the holding chamber-containing brain slices before recording. DARs antagonists and BD1063 were applied at least 20-25 min before application of psychostimulants. When basal effects of these antagonists were assessed, they were applied for at least one hour in the incubation chambers before recording. All cells were recorded within one hour from the moment the slices were removed from drug-containing incubation chambers.

To measure neuronal firing, whole-cell current-clamp recordings were performed with electrodes (3–5 MΩ, and 6-7 MΩ for Figure 4b and S5b) containing (in mM) 120 K-gluconate, 20 KCl, 10 HEPES, 0.2 EGTA, 2 MgCl2, 4 Na2ATP, and 0.3 Tris–GTP at a pH of 7.20-7.25. Data were filtered at 5 KHz, digitized at 10 kHz, and collected and analyzed using Clampex 10.5 software (Clampex 10.5.0.9, Molecular Devices, Inc.). Membrane potentials were maintained at – 80 mV, series resistances (10–18 MΩ) and input resistances were monitored on-line with a 40 pA current injection (150 msec) given before each 700 msec current injection stimulus. Only cells with a stable *R*i (Δ < 10%) for the duration of the recording were kept for analysis. Firing rate represents the average value measured from 2 to 4 cycles (700 ms duration at 0.1 Hz, –80 to +280 pA range with a 40 pA step increment, every 15 s). To avoid firing rundown in experiment presented in Figure 4 and Supplementary Figure S5b (intracellular cocaine), only two 700 msec current injection stimuli were given, at 200 pA and 240 pA with 15 s interval.

For experiments in Supplementary Figure S1, voltage-clamp protocols to measure Na+ currents consisted of a voltage-step command from a holding potential of -80 mV. The neuron was depolarized to -40 mV and -30 mV for 25 ms, every 30 s. Recordings were performed in ACSF containing picrotoxin (100 μM), TEA (5 mM), and CdCl2 (0.1 mM) to block GABA_A_R-mediated transmission, K+ channels, Ca2+ channels, and Ca2+-activated K+ channels respectively. To measure Na+ current whole-cell voltage-clamp recordings were performed with electrodes (3–5 MΩ) containing (in mM) 117 cesium gluconate, 2.8 NaCl, 20 HEPES, 0.4 EGTA, 5 tetraethylammonium-Cl, 2 MgATP, and 0.3 MgGTP, pH 7.2– 7.4 (285–295 mOsm). P/N 4 leak subtraction protocol was used, and Rs compensated at 60 %. To avoid possible drug contamination of other cells, only one cell per brain slice was recorded. The peak current amplitude for each voltage command was calculated from the average of 3-5 traces.

**Immunoprecipitation from Tissue**

Brain tissues were homogenized in ice-cold sucrose buffer 100 μl (0.32 M sucrose, 50 mM HEPES-KCl, pH 7.4, protease inhibitor cocktails (Sigma); with a glass Dounce homogenizer (40 strokes). After centrifugation at 900 g for 5 min, supernatants were transferred and collected into tubes. Pellets were homogenized with a sucrose buffer (100 μl) followed by centrifugation at 900 g for 5 min. The obtained supernatants were combined with those from the first centrifugation and protein concentration was measured using a protein assay kit (Bio-Rad). One hundred mg of protein from supernatants in 100 µl of sucrose buffer were mixed with an equal volume of 2x lysis buffer (100 Mm Tris-HCl, pH 7.4, 300 mM NaCl, and protease inhibitors, 1% NP-40) and incubated at 4°C for 30 min. Lysates were centrifuged at 12,000 x g for 15 min and the resultant supernatants were immunoprecipitated with the anti-Kv1.2 antibody (1 μl, overnight incubation) (Millipore Cat. MABN77 or NeuroMab Cat. 75-008). Immunoprecipitants were washed 4 times with 500 μl of 1 x lysis buffer and once with buffer without detergent. Samples were boiled in 30 μl of Laemelli buffer at 95C for 5 min. Immunoprecipitated proteins were analyzed by western blot using anti-Kv1.2, and anti-*σ*1 antibody to detect endogenous *σ*1 in brain samples. The densitometry readings of *σ*1 blots were normalized against the optical density of Kv1.2.

**Membrane Isolation**

Brain tissue was washed with ice-cold saline solution and homogenized with a prechilled teflon-glass homogenizer in 200 μl of homogenization buffer (10 mM HEPES, complete protease inhibitors cocktail, Roche, pH 7.5). For cell culture experiments, HEK293T cells (HEK-293 from ATCC, American Type Culture Collection) where homogenized in homogenization buffer by passing 7 times through 25 gauge needle attached to a 1 ml syringe. The samples were centrifuged at 600 x g for 5 min, the pellet was washed with 1 mL of homogenization buffer and centrifuged again at 600 x g for 5 min. Supernatants from both centrifugations were mixed and centrifuged at 20,000 x g for 20 min. The resulting pellet was suspended in 100 μl of homogenization buffer. Solution was added drop by drop to the pellet with vigorous shaking in order to prevent membrane agglutination and subsequent contamination of the membranes. Samples were mixed with 50 μl of binding buffer. 100 μl of beads (BioMag®Plus Concanavalin A) per sample were washed 3 times with binding buffer and the samples were added to the beads. Sample was incubated for 2 hrs and the beads were washed with 10 mM HEPES 5 times. The buffer was completely removed from beads and 50 μl of elution buffer (0.25 M methyl α-D-mannoside in TBS with 1% Triton X100) was added to the beads and incubated for 30 min at room temperature. Beads were removed from eluted proteins and 5 μl of 6x Laemmli buffer was added before being analyzed by western blot. For total and plasma membrane (Figure 3c, 3d), densitometry readings of Kv1.2 and *σ*1 blots were normalized against actin and to the average of their respective SAL group. Plasma membrane extraction was verified by the enrichment of the fraction with plasma membrane marker Na+/K+ ATPase pump and the absence of GRP78 (a marker for ER membranes), consistent with the published method ([8-10](#_ENREF_8)) .

**Immunoprecipitation From Cell Culture**

100% confluent cells overexpressing *σ*1 and Kv1.2 *σ*1 and IP3R in 6 wells plate (1 well per sample) were washed twice with cold Phosphate Buffered Saline (PBS) and treated with 0.25 mM of Dithiobis-succinimidyl-propionate (DSP, Thermo) to perform a chemical crosslinking of cellular proteins. After 30 min of incubation, the crosslinking reaction was quenched with TBS (3 washes). Fixed cells were then lysed in 0.3 ml of IP buffer (50 Mm Tris-HCl, pH 7.4, 150 mM NaCl, 1% Triton X-100, 0.01% SDS, and protease inhibitors), passed 7 times through 25 gauge needle attached on a 1 ml syringe and incubated for 30 min. Protein concentration of cellular extracts was measured using a Biorad protein assay kit. 160 mg of proteins from supernatants were mixed with IP buffer in equal volume. Cell lysates were immunoprecipitated with 1 μl anti-Kv1.2 antibody for all experiments except for Figure S4b where anti-V5 antibody (3 μl) was used to pull-down *σ*1. Antibodies were incubated overnight with the cell lysate. 25 μl (or 40 μl for V5 IP) of prewashed magnetic protein G beads were then applied, and samples were rotated for 2hrs at 4C. Beads were washed twice with 1 ml of IP buffer, and twice with TBS with Triton X100 0.5%. Samples were heated in 30 μl of Laemelli sample buffer at 95°C for 5 min. Proteins were analyzed by SDS-PAGE. The densitometry readings of *σ*1 blots were normalized against the optical density of Kv1.2. Immunoprecipitated proteins were analyzed by western blot using anti-Kv1.2, and anti-V5 to detect *σ*1 in HEK293T cells.

**Western Blotting**

To quantify *σ*1 and Kv1.2 total expressions in microdissected sections, tissue samples were solubilized in lysis buffer (50 mM Tris pH7.4, 150 mM NaCl, 0.1% SDS, 1% Triton X-100) and 30 μg of total protein lysate were analyzed. To measure total expression in cell culture, 10 μg of protein were separated from the samples for immunoprecipitation before adding the antibody.

Samples to measure total protein as well as immunoprecipitated and plasma membrane extracts, prepared as described above, were mixed with Laemelli buffer plus DTT, heated at 95°C for 5 min and separated in 4-15% SDS–PAGE. Proteins were electrophoretically transferred to nitrocellulose membranes. Membranes were blocked for 2 h at room temperature in TBS-T buffer containing 5% BSA. Membranes were incubated overnight with primary antibodies: anti-Kv1.2 (Millipore Cat. MABN77 or NeuroMab Cat. 75-008), Anti-Sigma 1R (Santa Cruz Biotechnology sc-137075) anti-Actin (Millipore Cat MABT219), anti-V5 (BioRad Cat MCA1360GA), anti-Sodium Pump Subunit alpha-2 (Millipore Cat. MCA1360GA), rabbit anti-IP3R (T443) (Jackson Immunoresearch), washed (4 times/5 min) in TBS-T with 1% BSA, and incubated for 1 h with Goat anti mouse or rabbit IRDye-conjugated secondary antibody (Li-cor cat. 92668170 and 82708365). Near infra-red fluorescence was captured using the Odyssey LI-COR Imaging Systems. Samples were excluded only when a western blot defect was observed.

**Statistics**

**Electrophysiology.** On recording days, data were collected from two drug-treated groups in semi-randomized manner. However, control cells (incubated in ACSF with vehicle) were recorded more often to ensure that control neuronal firing remained stable throughout experimental days, which explain small SEM for control groups. Recordings and analyzes were performed blind of the treatment, except for Figure 4a, S1a, and S4a, in which cocaine perfusion had to be controlled by the experimenter. Sample sizes are similar to standard electrophysiological studies conducted with rigor and reproducibility, i.e., were chosen so that differences between group can be interpreted in unequivocal manner (as performed in our previous studies ([1-3](#_ENREF_1)).

**Protein assays.** Samples from brain tissue were run and analyzed blind of the treatment, but not samples from HEK293T cells. For cell culture studies, every independent sample was prepared on different days (independent experiment) from different passages.

**In vivo drug treatments for protein assays and electrophysiological recordings**. Mice were randomly assigned to experimental groups, counter-balancing treatments per each cage so that mice housed together were not receiving the same treatment. No experimental group was tested without a matching control done simultaneously on mice of the same strain, age and sex (all experiments were performed on male mice).

**Supplementary Figure 1. Cocaine (3 μM) does not alter Na^+^ current and decreases neuronal firing of NAcSh D1R-MSNs in dose-dependent manner.** (**a**) Peak Na^+^ current density before and during cocaine (COC, 3 μM) (n = 8 cells/3 mice); currents were evoked by depolarizing test pulses (inset in **b**) to -40 mV and -30 mV for 25 ms, every 30 s. (**b**) Examples of Na^+^ current traces in ACSF and during bath application of cocaine for more than 10 min. Time was measured from the moment cocaine entered the recording chamber. Each data point represents an individual neuron. Calibration: 1 ms, 2 nA. Paired t-test when depolarized at -40 mV or -30 mV: p>0.05. (**c**) Left: Action potential (AP) trace depicting threshold (Thr) and amplitude (Ampl) measurement. Right: Cocaine (3 μM, 1 hr) does not alter AP amplitude (SAL, n = 12 cells/4 mice; COC, n = 15 cells/8 mice). AP amplitudes were obtained from the first spike evoked by the minimal depolarizing current pulse in every NAc neuron recorded in Figure 1c. AP amplitudes represent the difference between AP threshold and peak. Unpaired t-test, p>0.05. Hash marks indicate group means ± SEM. (**d**) Summary histogram from Figure 1**e** showing the number of spikes elicited at 240 pA for all groups (SAL, and COC at 0.2, 0.5, 1.0, and 3.0 μM) (SAL, n = 21 cells/8 mice; COC 0.2 μM, n = 11 cells/5 mice; COC 0.5 μM, n = 16 cells/5 mice; COC 1 μM, n = 9 cells/3 mice; COC 1 μM, n = 12 cells/8 mice). One-way ANOVA: ****p < 0.0001. Post hoc tests: SAL group is different from all COC groups except COC 0.2 μM: *p < 0.05, **p < 0.01, ****p < 0.0001. Data are represented as mean ± SEM.

**
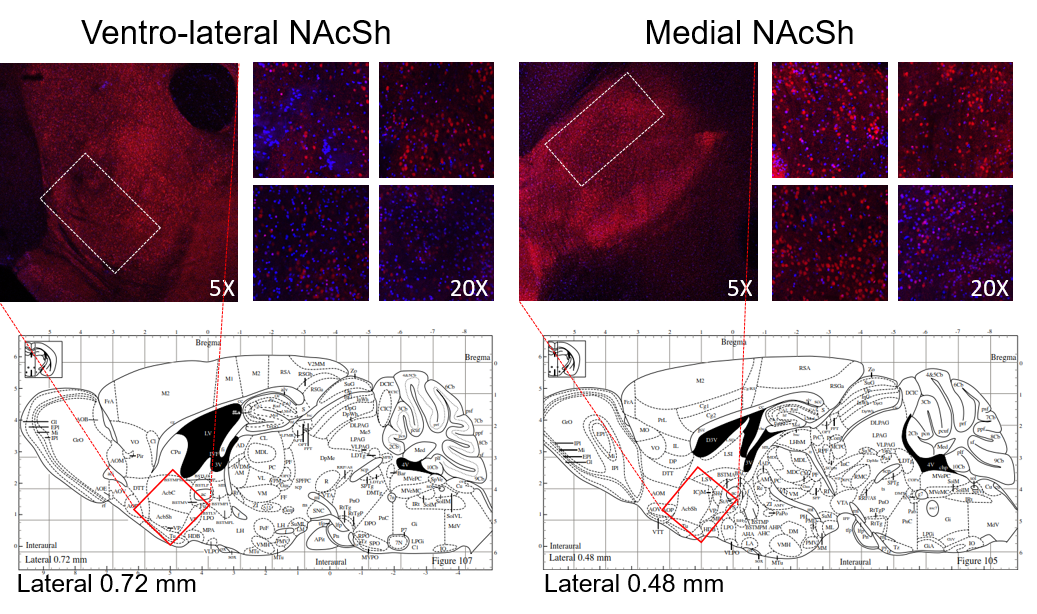
**

**Supplementary Figure 2. D1R-MSNs are not distributed homogenously throughout the NAc shell, and medial NAcSh contain high proportion of D1R-MSNs.** Left: Ventro-lateral NAcSh shown at 5X with examples of pictures taken at 20X in the dotted white selection. Right: Medial NAcSh shown at 5X with examples of pictures taken at 20X (subregion where recordings are performed). In red, Drd1a-tdTomato expressing cells (D1R-MSNs); in blue, DAPI. Bottom (left and right): Corresponding figures taken from the mouse brain atlas ([7](#_ENREF_7)).

**Supplementary Figure 3. Cocaine-induced FRD in NAcSh D1R-MSNs is not prevented by DA receptor antagonists.** Complete input-output curves from data represented in Figure 2a. SAL and COC groups in (**a**), (**b**), (**c**), and (**d**) are the same; however, for the sake of visual clarity and visual group comparisons, they are represented in separate graphs. In (**a**), (**b**), (**c**), and (**d**): although experimental groups are represented in different graphs, cells where recorded in parallel, therefore, statistical analyses were performed on all groups combined. Two-way ANOVA, ****p < 0.0001. Post-hoc tests showed that SAL is different from all groups with COC, and not different from SCH, SULP, or SCH + SULP groups. ****p<0.0001, ***p < 0.001. (SAL, n = 17 cells/9 mice; COC, 9 cells/4mice; SCH, 8 cells/3 mice; SCH + COC, n = 8 cells/3 mice; SULP, n = 12 cells/6 mice; SULP + COC, 9 cells/3mice, SULP + SCH, 10 cells/3 mice; SULP + SCH + COC, 9 cells/4 mice). Data are represented as mean ± SEM.


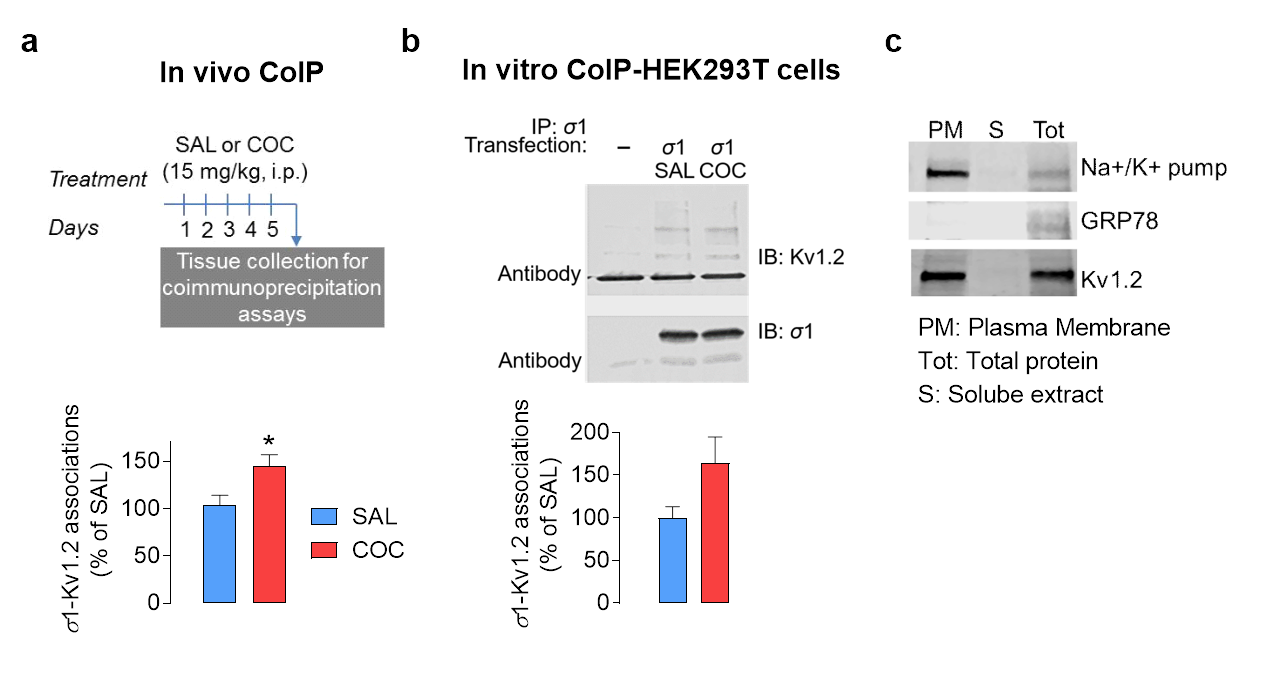


**Supplementary Figure 4. In vivo cocaine enhances *σ*1-Kv1.2 interactions.** (**a**) Top: Experimental timeline. Bottom: Cocaine (five times, once-daily, 10-15 mg/kg, intraperitoneal, i.p.) enhances *σ*1-Kv1.2 CoIP in NAcSh (3 samples/group, NAcSh from 5 mice/sample). Cell lysates were immunoprecipitated with the anti-Kv1.2 antibody; immunoprecipitated proteins were analyzed by western blot using anti-Kv1.2, and anti-*σ*1 antibody. (**b**) HEK293T cells expressing *σ*1–V5 and Kv1.2 were treated with saline or cocaine (3 μM, 1hr) and immunoprecipitated with the anti-V5 antibody; immunoprecipitated proteins were analyzed by western blot using anti-Kv1.2 (upper), and anti-V5 to detect *σ*1 (lower) (3 independent samples/group). (**c**) Total protein (Tot, lane three) or soluble extract (S, negative control, lane two) were incubated with ConA-beads to isolate plasma membrane (PM, lane one). Proteins eluted from beads were measured (2 μg) and analyzed by western blot (proteins from beads incubated with soluble extract were undetectable) and compared with 20 μg of total protein. Note that the PM lane contains the marker Na+/K+ pump marker but not the ER marker GRP78. In (**a**): Unpaired t-test, *p < 0.05. Data are represented as mean ± SEM.


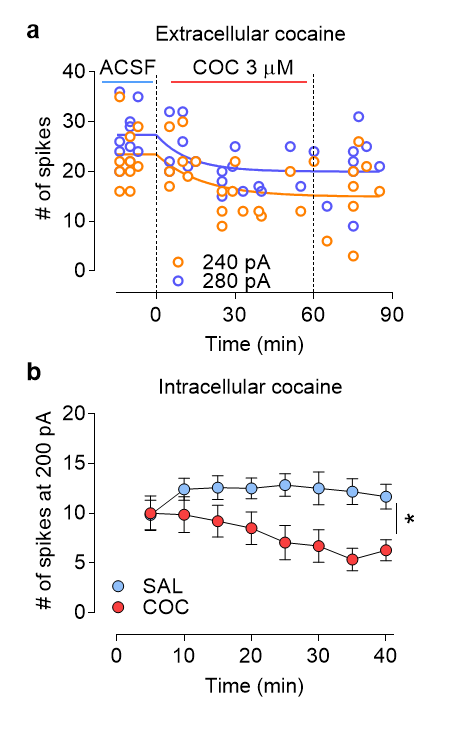


**Supplementary Figure 5. Cocaine-induced FRD in NAcSh D1R-MSNs is initiated by intracellular cocaine.** (**a**) Cocaine (3 μM) perfused in the recording chamber decreases firing rate elicited with 240 or 280 pA current injection within 30 min. Each data point represents individual neurons (n = 36 cells/8 mice). Nonlinear regression shown that cocaine-induced FRD fit an exponential with one phase decay. R^2^ for nonlinear fit for 240 and 280 pA are 0.2654 and 0.2705 respectively. (**b**) Mean number of spikes elicited at 200 pA for neurons recorded with micropipettes containing cocaine (COC, 3 μM) is decreased compared to vehicle (SAL) (SAL, n = 12 cells/7 mice; COC, n = 14 cells/9 mice). Data are represented as mean ± SEM.

**REFERENCES**

1. Kourrich S, Thomas MJ. Similar neurons, opposite adaptations: psychostimulant experience differentially alters firing properties in accumbens core versus shell. J Neurosci. 2009;29(39):12275-83.

2. Kourrich S, Hayashi T, Chuang JY, Tsai SY, Su TP, Bonci A. Dynamic interaction between sigma-1 receptor and Kv1.2 shapes neuronal and behavioral responses to cocaine. Cell. 2013;152(1-2):236-47.

3. Kourrich S, Klug JR, Mayford M, Thomas MJ. AMPAR-Independent Effect of Striatal αCaMKII Promotes the Sensitization of Cocaine Reward. J Neurosci. 2012: DOI:10.1523/JNEUROSCI.6391-11.2012.

4. Belleau ML, Warren RA. Postnatal development of electrophysiological properties of nucleus accumbens neurons. J Neurophysiol. 2000;84(5):2204-16.

5. Govindaiah G, Wang Y, Cox CL. Substance P selectively modulates GABA(A) receptor-mediated synaptic transmission in striatal cholinergic interneurons. Neuropharmacology. 2010;58(2):413-22.

6. Kreitzer AC. Physiology and pharmacology of striatal neurons. Annual review of neuroscience. 2009;32:127-47.

7. Paxinos G, Franklin KBJ. The Mouse Brain in Stereotaxic Coordinates. 2nd ed: Academic Press, San Diego; 2001.

8. Lee YC, Block G, Chen H, Folch-Puy E, Foronjy R, Jalili R, et al. One-step isolation of plasma membrane proteins using magnetic beads with immobilized concanavalin A. Protein expression and purification. 2008;62(2):223-9.

9. Lee YC, Liu HC, Chuang C, Lin SH. Lectin-Magnetic Beads for Plasma Membrane Isolation. Cold Spring Harbor protocols. 2015;2015(7):674-8.

10. Lee YC, Srajer Gajdosik M, Josic D, Lin SH. Plasma membrane isolation using immobilized concanavalin A magnetic beads. Methods in molecular biology. 2012;909:29-41.
